# Identification of prognostic factors and construction of nomogram to predict cancer‐specific survival for patients with ovarian granulosa cell tumors

**DOI:** 10.1002/cnr2.2046

**Published:** 2024-03-20

**Authors:** Yue Zhang, Zhen Zhang, Xinyao Ding, Keyi Zhang, Youren Dai, Wenjun Cheng, Chengyan Luo

**Affiliations:** ^1^ Department of Gynecology The First Affiliated Hospital of Nanjing Medical University Nanjing China; ^2^ Department of Gynecology Suqian First Hospital Suqian China

**Keywords:** cancer‐specific survival, nomogram, ovarian granulosa cell tumors, prognostic factors

## Abstract

**Background:**

Ovarian granulosa cell tumors (OGCTs) feature low incidence, indolent growth and late recurrence. Treatment for recurrent OGCTs is challenging.

**Methods:**

The present study was designed to explore the prognostic factors and establish a nomogram to predict cancer‐specific survival (CSS) for OGCTs patients. Enrolled in the study were 1459 eligible patients in the Surveillance, Epidemiology, and End Results (SEER) database, who were randomized to the training (*n* = 1021) or testing set (*n* = 438) at a ratio of 7:3. Univariate and multivariate Cox regression analyses were employed to screen the prognostic factors. The predictors were determined by using the Least absolute shrinkage and selection operator (LASSO) regression analysis. The model was constructed via the Cox proportional hazards risk regression analysis. The performance and clinical value of the nomograms was assessed with C‐index, calibration plots, and decision curve analysis.

**Results:**

Age, pTNM stage, tumor size, surgery of the primary tumor, surgery of regional lymph nodes (LNs), residual disease after surgery, and chemotherapy were considered as significant predictive factors for CSS in OGCTs patients. After screening, the prognostic factors except surgery of regional LNs and chemotherapy were employed to build the nomogram. With desirable discrimination and calibration, the nomogram was more powerful in predicting CSS than the American Joint Committee on Cancer staging system in clinical use.

**Conclusion:**

This novel prognostic nomogram, which comprises a stationary nomogram and a web‐based calculator, offers convenience for clinicians in personalized decision‐making including optimal treatment plans and prognosis assessments for OGCTs patients.

## 1 INTRODUCTION

Ovarian granulosa cell tumors (OGCTs), as the most prevalent sex cord‐stromal tumors (SCSTs), making up 2% to 5% of all ovarian malignancies.^1^ According to the fifth edition World Health Organization (WHO) classification of female genital tumors, OGCTs encompass adult ovarian granulosa cell tumors (aOGCTs) and juvenile ovarian granulosa cell tumors (jOGCTs), representing 95% and 5%, respectively.^2^ OGCTs are characterized by slow progression and relatively favorable prognosis, with 78–91% of women being detected at early‐stage, and its 5‐year overall survival (OS) rate ranging between 75%–95%.^1^, ^3^ This suggests that OGCTs exhibit an indolent character. Nonetheless, 20%–30% of them still relapse after treatment, some even after 10 years or more.^4^ Recurrence of OGCTs frequently involves multiple sites, including the pelvis, liver, diaphragm, bowel, and para‐aortic lymph nodes.^5^ Since OGCTs present non‐sensitive to chemotherapy, surgery is listed as mainstay treatment for primary, as well as relapsed and metastatic OGCTs in National Comprehensive Cancer Network (NCCN) guidelines.^6^, ^7^ With the increase of relapse frequency, surgical intervention became extremely difficult owing to extensive adhesion and tumor invasion. Therefore, the treatment for recurrent disease is challenging and 70% of women with relapse eventually die of the disease.^8^ Though some cohort studies have demonstrated the risk factors for relapse of OGCTs,^9^, ^10^ few literatures are available on the factors predicting the survival outcomes and on a large populations‐based predictive model for OGCTs. Therefore, the present study, exploiting the Surveillance, Epidemiology, and End Results (SEER) database, was designed to dig out the prognostic factors of cancer specific survival (CSS) and construct an individualized nomogram that can predict of 5‐ and 10‐year CSS among OGCTs patients, which may provide clinicians with valuable evidence on decision‐making, especially regarding optimal treatment plans and personalized prognosis.

## 2 MATERIALS AND METHODS

### 2.1 Data source and variables

All the data was retrieved from the SEER database of the National Cancer Institute, an information source for cancer incidence and survival data in the United States, which updated annually and encompassed 35% of the United States population from 17 registries (Incidence‐SEER Research Plus Data, 17 Registries, Nov 2021 Sub (2000–2019)). The SEER database provides sufficient patients and complete follow‐up information for malignant tumors, including OGCTs, making it an important source of research. The data was collected using SEER*Stat 8.4.0 software, accessible at http://www.seer.cancer.gov.^11^ Inclusion criteria included the following: (1) patients with histopathologically confirmed diagnoses of aOGCTs and jOGCTs: International Classification of Diseases for Oncology, third edition (ICD‐O‐3) with histology/behavior codes 8620/3 (aOGCTs), 8622/3 (jOGCTs), and a site code of C56.9, (2) patients with a first primary tumor that was OGCTs, and (3) patients with a known survival time and a known cause of death. Histologically, aOGCTs are neoplasms with diverse morphology consisting of granulosa cells growing mixed with varying numbers of fibroblasts or papillary cells, whereas jOGCTs are another histological subtype consisting of primitive granulosa cells that grow in solid and follicular forms.^2^ Based on the coding, this study retrieved 1545 patients diagnosed with OGCTs between 2000 and 2019. The following criteria were used for exclusion: (1) patients diagnosed by autopsy or death certificate, (2) patients with unknown American Joint Committee on Cancer (AJCC) stage, (3) patients with unknown survival time, (4) patients with a survival time of less than 1 month, and (5) patients with unknown cause of death. A total of 86 patients were excluded following the above exclusion criteria. The variables obtained from the SEER database included age at diagnosis, race, marital status, tumor size, presence of multi‐primary tumors, AJCC stage, pT stage, pN stage, pM stage, surgery of the primary tumor, surgery of regional LNs, surgery of distant metastasis, presence of residual disease after surgery, administration of chemotherapy, administration of radiation, cause‐specific survival time, and survival status. Age at diagnosis and tumor size were non‐normally distributed, and then were categorized based on the cutoff values calculated with x‐tile software (version X‐tile 3.6., Yale University, New Haven, Connecticut, USA).^12^ Age at diagnosis were stratified as ≤64 years old and >64 years old. Tumor sizes were categorized into ≤99 and >99 mm. For the cohort, 206 (14.1%) cases presented with unknown residual disease after surgery and 312 (21.4%) cases with unknown tumor size. For the missing data, the variables were tested being missed at random and multiple imputation by chained equation (MICE) package was performed 20 cycles to control bias.^13^

### 2.2 Endpoints

**FIGURE 1:**
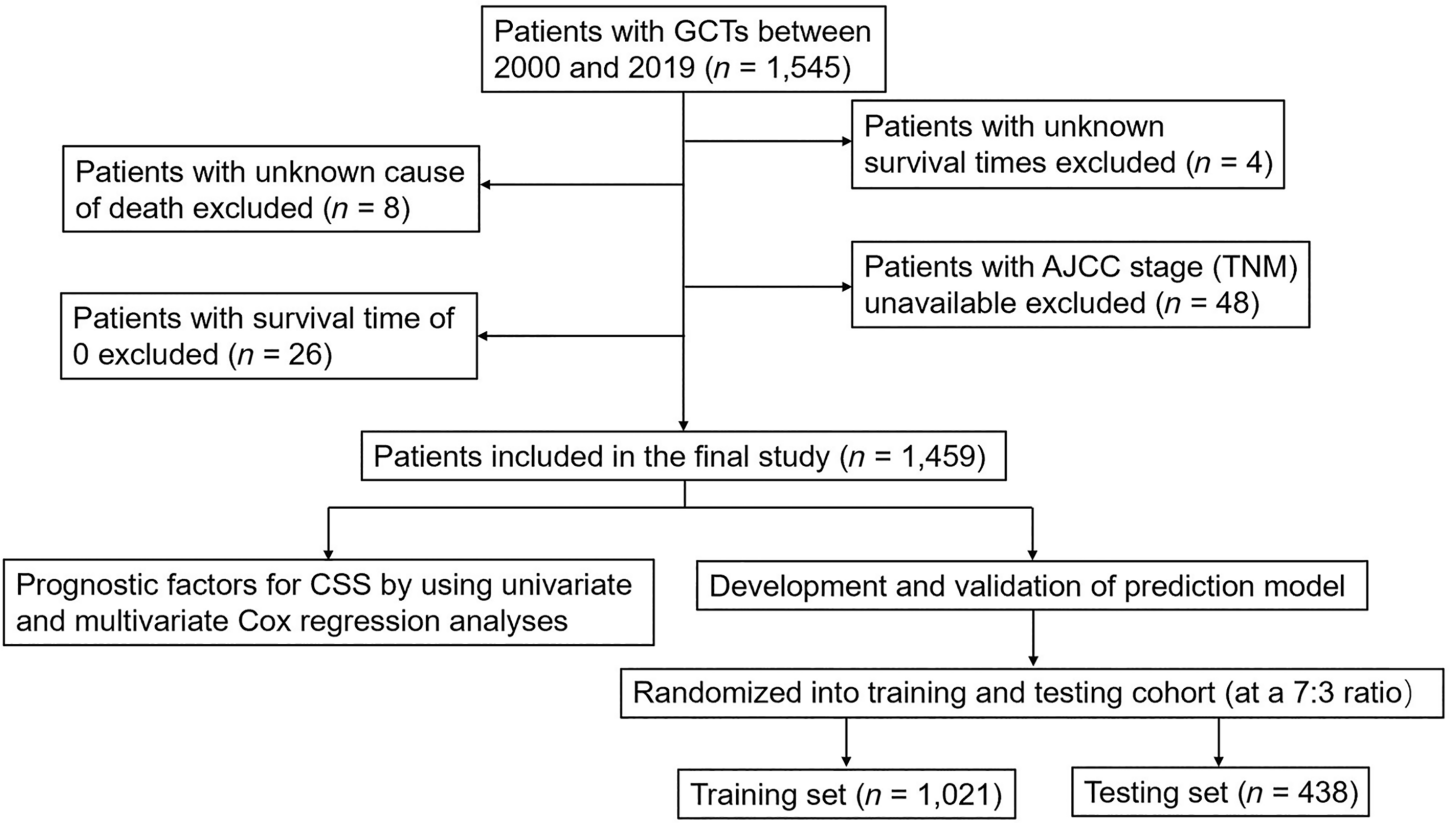
Flowchart of data process for this study.

CSS was used as a primary endpoint in this study because OGCTs were low‐grade malignant tumors with good prognosis. Yet as the course prolongs, the overall survival (OS) outcomes for OGCT patients were compromised not only by patients' age, but by tumors other than OGCTs. The CSS time referred to the duration from the diagnosis to either the date of death caused by OGCTs or the last day of follow‐up. After screening, 1459 women with OGCTs were enrolled. The censor time point of the present study was December 31, 2021, the most recent update of the follow‐up time. Figure 1 shows the data processing procedure.

### 2.3 Variables selection

For the whole cohort (*n* = 1459), all the variables except AJCC stage were incorporated in the univariate Cox regression analysis to screen factors related to CSS. Subsequently, a multivariable analysis was conducted for the significant factors (*p* < .05). The hazard ratio (HR) and the estimated 95% confidence interval (CI) were determined and presented using a forest plot. The multivariate analysis indicated age (*p* < .001), pT stage (*p* = .013), pN stage (*p* = .041), pM stage (*p* < .001), tumor size (*p* < .001), surgery of primary tumor (*p* = .008), surgery of regional LNs (*p* = .006), residual disease after surgery (*p* < .001), and chemotherapy (*p* = .018) as independent predictive factors for CSS (Table 2).

### 2.4 Model development and validation

Through univariate analysis and multivariate analysis, nine variates were recognized as independent predictive factors for CSS. The whole cohort with OGCTs (*n* = 1459) were randomized into the training (*n* = 1021) or testing set (*n* = 438) in a 7:3 ratio. To screen the most important variables enrolled in the model, least absolute shrinkage and selection operator (LASSO) regression analysis via 10‐fold cross‐validation was used in the training set.^14^ The LASSO regression analysis penalized the absolute values of the regression coefficients of the variables. As the penalties increases, the estimates of the factors that have a weak effect on CSS shrank toward zero, ultimately retaining the most effective predictors associated with CSS in the model. Consequently, after screening the variables by using the LASSO shrinkage technique, it prevents overfitting of the model, simplifies the model, and facilitates its application. After integrating the predictive power and clinical significance of the variables, we incorporated seven factors, including age, pT stage, pN stage, pM stage, tumor size, surgery of the primary tumor, and residual disease after surgery, into the model. The model was constructed by employing Cox proportional hazards regression analysis with training set and validated with testing set. Meanwhile, we assessed the proportional hazards assumption among the covariates in the model by examining influential case, multicollinearity, variance inflation factor (VIF) (data not shown). Based on the model, static and dynamic nomogram for 5‐ and 10‐year CSS were further developed as visual representations, respectively.

### 2.5 Model evaluation and application

The nomograms' power was quantified with concordance index (C‐index), indicating discrimination of the model. In addition, its calibration ability was evaluated with calibration plots, which indicated the consistency between the predicted and actual probabilities. To evaluate the feasibility and validity of the predictive model, the prognostic scores of all participants in the cohort were calculated based on the model. The patients from both the training and testing sets were classified into low‐ and high‐risk groups according to the median values of prognostic scores in the training set. Kaplan–Meier survival curves of low‐ and high‐risk groups were obtained and analyzed via log‐rank test. To assess the practicality of this model in clinical settings, we employed decision curve analysis (DCA) to calculate the net benefits (NB) at various threshold probabilities for clinical decision‐making and compared the model with the AJCC staging system.^15^

### 2.6 Statistical analyses

All statistics were processed by R software (version 4.0.4, http://www.r-project.org/). The R packages MatchIt, mice, survival, survminer, pROC, rms, foreign, caret, MASS, car, ggdca, dcurves, and DynNom were used for the analysis. The continuous variables were not normally distributed and denoted by median and interquartile range values (IQR). Categorical variables were represented by frequencies and percentages. Chi‐square test or Fisher's exact test was conducted for differences among categorical variables, and Mann–Whitney *U* test or Kruskal–Wallis *H* test for the differences among continuous variables, when appropriate. Prognostic factors for the patients' CSS were investigated by univariate and multivariate Cox regression analysis. LASSO regression analysis via 10‐fold cross‐validation was employed to screen predictors for CSS. The predictive model was generated with Cox proportional hazards risk regression analysis on the training set and validated on the testing set. The model's clinical usefulness and NB were analyzed via DCA. Survival probabilities were calculated by employing the Kaplan–Meier method and log‐rank test. Flowchart of the research design and data analysis was shown in Figure S3. The *p* values less than .05 (two‐tailed tests) were judged as statistically significant.

## 3 RESULTS

### 3.1 Patients' clinicopathologic features

Of 1459 eligible OGCT patients enrolled in the present study, 1427 (97.8%) cases had aOGCTs and 32 (2.2%) had jOGCTs. The patients' median age at diagnosis was 51 (IQR: 41–60) years. In terms of disease stage, 995 (68.1%) patients were diagnosed with stage I disease, while 204 (14.0%), 108 (7.4%), and 80 (5.4%) were diagnosed with stage II, III, and IV. The median tumor size was 90 (IQR: 50–155) mm. Regarding surgical approaches, 1380 (94.6%) patients received surgery for primary site, 634 (43.5%) patients had surgery for regional LNs and 151 (10.3%) patients underwent surgery for metastatic tumor. For adjuvant therapy, 421 (28.9%) cases received chemotherapy and 21 (1.4%) cases were treated with radiation. In the raw data before missing data handled, complete resection was achieved in 1131 (77.5%) patients, while residual tumors after surgery were left in 172 (11.8%) patients and unknown data in 206 (14.1%) patients. Of the patients, 614 (42.1%) and 533 (36.5%) had a tumor size of ≤99 and >99 mm, respectively. The median time of follow‐up was 127 (IQR: 62–189) months in the whole population. The OGCTs‐specific survival rates for 5‐ and 10‐year were 61.3% and 31.6%, respectively. To develop and validate the nomogram, the whole cohort with OGCTs (*n* = 1459) were randomized to either the training set (*n* = 1021) or the testing set (*n* = 438) at a ratio of 7:3. The patients of the training and testing sets presented comparable demographic and clinical‐pathological characteristics (*p* > .05), as shown in Table 1. Table S1 shows the details of the two groups of patients after processing the missing data.

**TABLE 1:** Demographic and clinicopathologic characteristics in patients with OGCTs from the SEER database.

Variable	Training set (*N* = 1021)	Testing set (*N* = 438)	*p* value
Age (years)			.768
≤64	831 (81.4%)	360 (82.2%)	
>64	190 (18.6%)	78 (17.8%)	
Race (%)			.459
White	696 (68.2%)	295 (67.4%)	
Black	240 (23.5%)	98 (22.4%)	
other	85 (8.3%)	45 (10.3%)	
Marital status (%)			.658
Single	261 (25.6%)	116 (26.5%)	
Married or ever married	705 (69.0%)	294 (67.1%)	
unknown	55 (5.4%)	28 (6.4%)	
Multi‐primary tumors (%)			.547
One primary only	839 (82.2%)	359 (82.0%)	
1st of 2 or more primaries	80 (7.8%)	29 (6.6%)	
2nd or more of primaries	102 (10.0%)	50 (11.4%)	
AJCC stage (%)			.858
I	689 (67.5%)	306 (69.9%)	
II	128 (12.5%)	52 (11.9%)	
III	146 (14.3%)	58 (13.2%)	
IV	58 (5.7%)	22 (5.0%)	
pT Stage (%)			.644
T1	708 (69.3%)	314 (71.7%)	
T2	142 (13.9%)	54 (12.3%)	
T3	171 (16.7%)	70 (16.0%)	
pN Stage (%)			.941
N0	993 (97.3%)	427 (97.5%)	
N1	28 (2.7%)	11 (2.5%)	
pM Stage (%)			.650
M0	962 (94.2%)	416 (95.0%)	
M1	59 (5.8%)	22 (5.0%)	
Surgery of primary tumor (%)			.417
No	59 (5.8%)	20 (4.6%)	
Yes	962 (94.2%)	418 (95.4%)	
Surgery of regional LNs (%)			.477
No	584 (57.2%)	241 (55.0%)	
Yes	437 (42.8%)	197 (45.0%)	
Surgery of distant metastasis (%)			.552
No	919 (90.0%)	389 (88.8%)	
Yes	102 (10.0%)	49 (11.2%)	
Chemotherapy (%)			.385
No	719 (70.4%)	319 (72.8%)	
Yes	302 (29.6%)	119 (27.2%)	
Radiation (%)			.566
No	1008 (98.7%)	430 (98.2%)	
Yes	13 (1.3%)	8 (1.8%)	
Tumor size (mm)			.560
≤99	427 (41.8%)	187 (42.7%)	
>99	380 (37.2%)	153 (34.9%)	
Missing	214 (21.0%)	98 (22.4%)	
Residual disease after surgery (%)			.093
No	769 (75.3%)	362 (82.6%)	
Yes	92 (9.0%)	30 (6.8%)	
Missing	160 (15.6%)	46 (10.5%)	
CSS			.938
Alive	889 (87.1%)	380 (86.8%)	
OGCTs specific death	132 (12.9%)	58 (13.2%)	

Abbreviations: AJCC, American Joint Committee on Cancer; CSS, cancer‐specific survival; LNs, lymph nodes; OGCTs, ovarian granulosa cell tumors; pM stage, stage of metastasis; pN stage, stage of lymph nodes; pT stage, stage of primary tumor; SEER, Surveillance, Epidemiology, and End Results.

### 3.2 Prognostic factors for CSS

**FIGURE 2:**
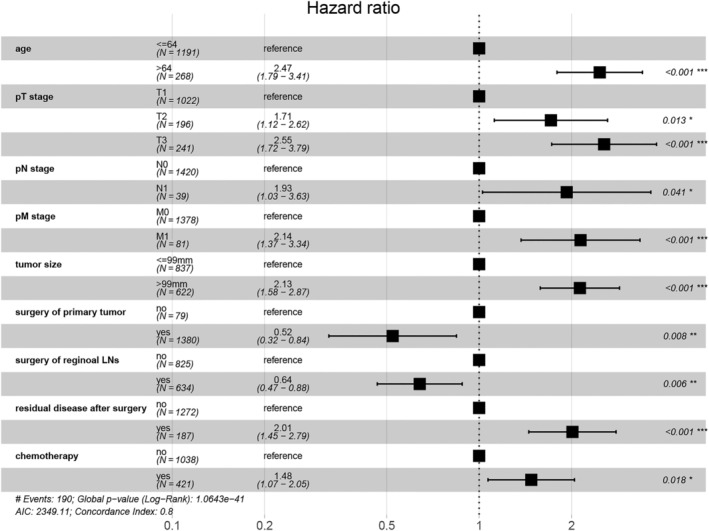
Forest plot demonstrating the prognostic factors for CSS in the whole population with OGCTs by using multivariate Cox regression analysis. CSS, cancer‐specific survival; OGCTs, ovarian granulosa cell tumors; LNs, lymph nodes; pM stage, stage of metastasis; pN stage, stage of lymph nodes; pT stage, stage of primary tumor.

The CSS‐associated prognostic factors for the whole population were determined by using the univariate and multivariate Cox regression analyses. The results were presented in Table 2. Univariate analysis indicated age at diagnosis, pT stage, pN stage, pM stage, tumor size, surgery of primary tumor, surgery of regional LNs, chemotherapy, and residual disease after surgery exhibited significant association with CSS (all *p* < .05). The factors were further enrolled into the multivariate analysis, which revealed that the patients presented poorer prognosis with older age (>64 years) (HR = 2.47, 95% CI = 1.79–3.41, *p* < .001), advanced pT stage (pT stage II, HR = 1.7, 95% CI = 1.12–2.62, *p* = .013 and pT stage III, HR = 2.55, 95% CI = 1.72–3.79, *p*<.001), pN1 stage (HR = 1.93, 95% CI = 1.03–3.63, *p* = .041), pM1 stage (HR = 2.14, 95% CI = 1.37–3.34, *p* < .001), tumor size (>99 mm) (HR = 2.13, 95% CI = 1.58–2.87, *p* < .001), residual disease after surgery (HR = 2.01, 95% CI = 1.45–2.79) and chemotherapy (HR = 1.48, 95% CI = 1.07–2.05) were independent risk factors of CSS, whereas the women receiving surgery of the primary site (HR = 0.52, 95% CI = 0.32–0.84, *p* = .008) and surgery of regional lymph nodes (LNs) (HR = 0.64, 95% CI = 0.47–0.88, *p* = .006) showed improved prognosis. The forest plot depicted the results of multivariate Cox regression analysis (Figure 2). The results showed nine variables, including age, pTNM stage, tumor size, surgery of primary tumor, surgery of regional LNs, residual disease after surgery, and chemotherapy, were independent predictors of CSS for OGCTs patients.

**TABLE 2:** Univariate and multivariate Cox hazards regression analysis on prognostic factors for CSS in the whole population with OGCTs.

Variable	Univariate analysis		Multivariate analysis			
	HR	95% CI	*p* value	HR	95% CI	*p* value
Age (years)						
≤64	1.000			1.000		
>64	2.960	2.180–4.010	<.001	2.469	1.790–3.411	<.001
Race						
White	1.000			1.000		
Black	1.320	0.954–1.810	.094			
Other	0.676	0.365–1.250	.214			
Marital status						
Single	1.000			1.000		
Married or ever married	0.808	0.585–1.120	.195			
Unknown	0.945	0.493–1.810	.864			
Multi‐primary tumors						
One primary only	1.000			1.000		
1st of 2 or more primaries	0.660	0.367–1.190	.165			
2nd or more of primaries	1.060	0.651–1.730	.812			
pT stage						
T1	1.000			1.000		
T2	2.730	1.830–4.070	<.001	1.715	1.123–2.620	.013
T3	5.750	4.190–7.880	<.001	2.553	1.724–3.794	<.001
pN stage						
N0	1.000			1.000		
N1	2.270	1.230–4.170	.009	1.925	1.003–3.631	.041
pM stage						
M0	1.000			1.000		
M1	5.070	3.420–7.500	<.001	2.138	1.370–3.343	.001
Surgery of primary tumor						
No	1.000			1.000		
Yes	0.214	0.141–0.326	<.001	0.524	0.324–0.840	.008
Surgery of regional LNs						
No	1.000			1.000		
Yes	0.553	0.409–0.747	<.001	0.643	0.471–0.876	.013
Surgery of distant metastasis						
No	1.000			1.000		
Yes	1.470	0.974–2.230	.067			
Chemotherapy						
No	1.000			1.000		
Yes	2.220	1.660–2.960	<.001	1.481	1.071–2.049	.018
Radiation						
No	1.000			1.000		
Yes	0.815	0.202–3.280	.774			
Tumor size (mm)						
≤99	1.000			1.000		
>99	1.940	1.450–2.590	<.001	2.016	1.451–2.971	<.001
Residual disease after surgery						
No	1.000			1.000		
Yes	3.650	2.720–4.890	<.001	2.055	1.479–2.855	<.001

Abbreviations: CI, confidence interval; CSS, cancer‐specific survival; HR, hazard ratio; LNs, lymph nodes; OGCTs, ovarian granulosa cell tumors; pM stage, stage of metastasis; pN stage, stage of lymph nodes; pT stage, stage of primary tumor.

### 3.3 Nomogram construction

**FIGURE 3:**
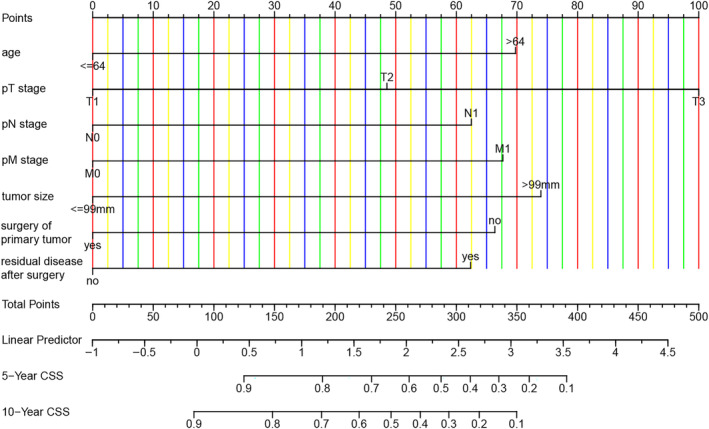
Nomogram for predicting 5‐ and 10‐year CSS in patients with OGCTs. CSS, cancer‐specific survival; OGCTs, ovarian granulosa cell tumors; pM stage, stage of metastasis; pN stage, stage of lymph nodes; pT stage, stage of primary tumor.

To simplify the model and to minimize overfitting risk, LASSO regression analysis with 10‐fold cross‐validation was used to determine the factors with closet relevance to CSS for the model (Figure S1). As a result, the five most important predictors with nonzero coefficient value were distinguished, including age, pT stage, pM stage, tumor size, and surgery of primary tumor. According to International Federation of Gynecology and Obstetrics (FIGO) staging system (2014) for ovarian cancer,^16^ tumor with retroperitoneal LNs involvement is classified as stage IIIA1 and presents worse prognosis compared to those in stage I or stage II.^10^ For ovarian neoplasms, complete resection without residual tumor remains important prognostic factor, which was proved in the previous study for women with OGCTs.^17^ Combined the predictive power with clinical significance of the variables, age, pTNM stage, tumor size, surgery of primary tumor, and residual disease after surgery were employed to develop the prediction model. We preformed Cox proportional hazards risk regression analysis to build the model with training set and validated it with testing set. During the procedure, the proportional hazards (PH) assumption among the covariates in the mode was evaluated, which demonstrated the variables met the PH assumption. After adjusting for confounders, the model, which includes survival time and OGCTs‐specific death as outcome variables, calculates the baseline survival at time *t* (S0(*t*)) and the regression coefficients for the included variables. The regression coefficients for these variables are shown below: age > 64 years equals 0.8079027, T2 stage equals 0.5618929, T3 stage equals 1.1585345, N1 stage equals 0.7238137, and M1 stage equals 0.7841176, tumor size >99 mm equals 0.8566873, surgical resection of primary tumor equals −0.7681007, and postoperative residual disease equals 0.7222439. Patients' CSS probability could be calculated by using the formula: S(*t*) = S0(*t*) ^exp (0.8079027* age >64 years + 0.5618929* stage T2 +1.1585345* stage T3 + 0.7238137* stage N1 + 0.7841176* stage M1 + 0.8566873* tumor size >99mm −0.7681007* surgery of primary tumor + 0.7222439* residual disease after surgery), where S0(*t*) was the mean CSS rate at time *t* (0.959, 0.948, and 0.4917, at three, five, and ten years, respectively). In order to estimate the patients' CSS probability more conveniently, static and dynamic nomograms for 5‐ and 10‐year CSS were further constructed as visual representations, respectively (Figure 3). The dynamic nomogram was presented with an online calculator (https://luochengyan66.shinyapps.io/dynnomapp/), where the patient's survival probability and 95% CI at a given timepoint could be derived by entering the value of the predictors and time. For example, a 37‐year‐old patient with OGCT, tumor stage T3, N0, M0, tumor size 130 mm, who underwent primary site resection with residual lesions postoperatively, had 5‐ and 10‐year CSS probabilities of 0.68 (95% CI: 0.58–0.80) and 0.54 (95% CI: 0.42–0.69), respectively, according to the predictive model. (Figure S2).

### 3.4 Performance of nomogram

**FIGURE 4:**
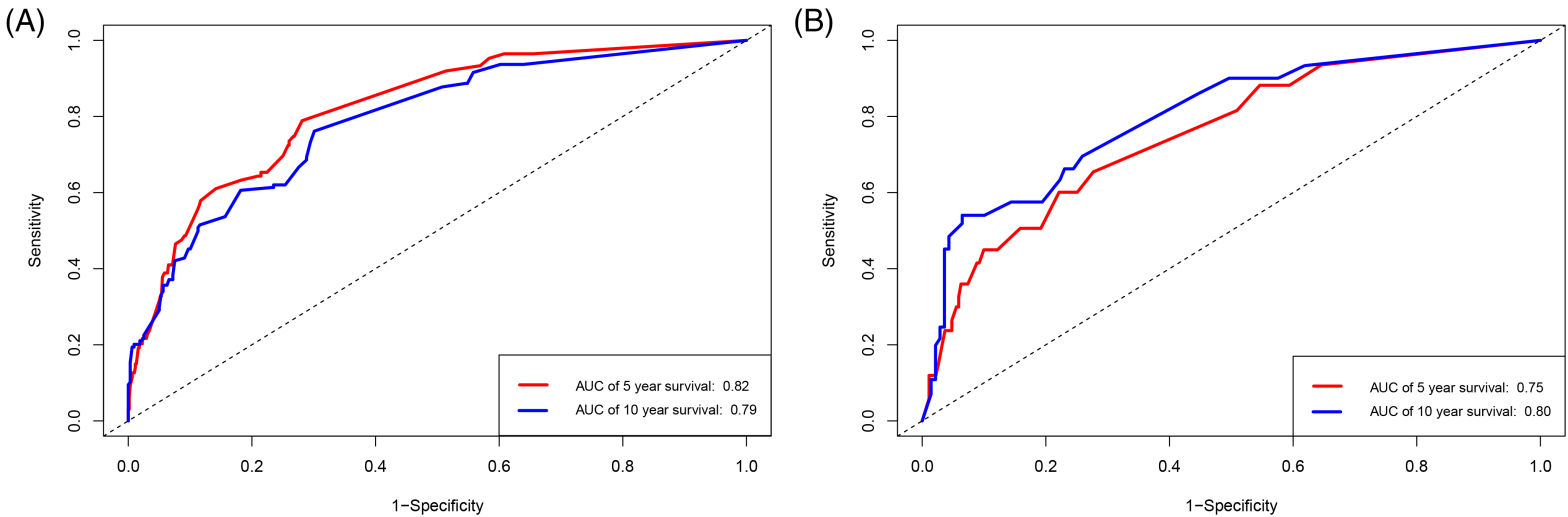
The ROC curves of the nomogram for 5‐ and 10‐year CSS in patients with OGCTs. (A) ROC curve for 5‐ and 10‐year CSS in the training set; (B) ROC curve for 5‐ and 10‐year CSS in the testing set. AUC, Area under ROC curve; CSS, cancer‐specific survival; OGCTs, ovarian granulosa cell tumors; ROC, receiver operator characteristic.

**FIGURE 5:**
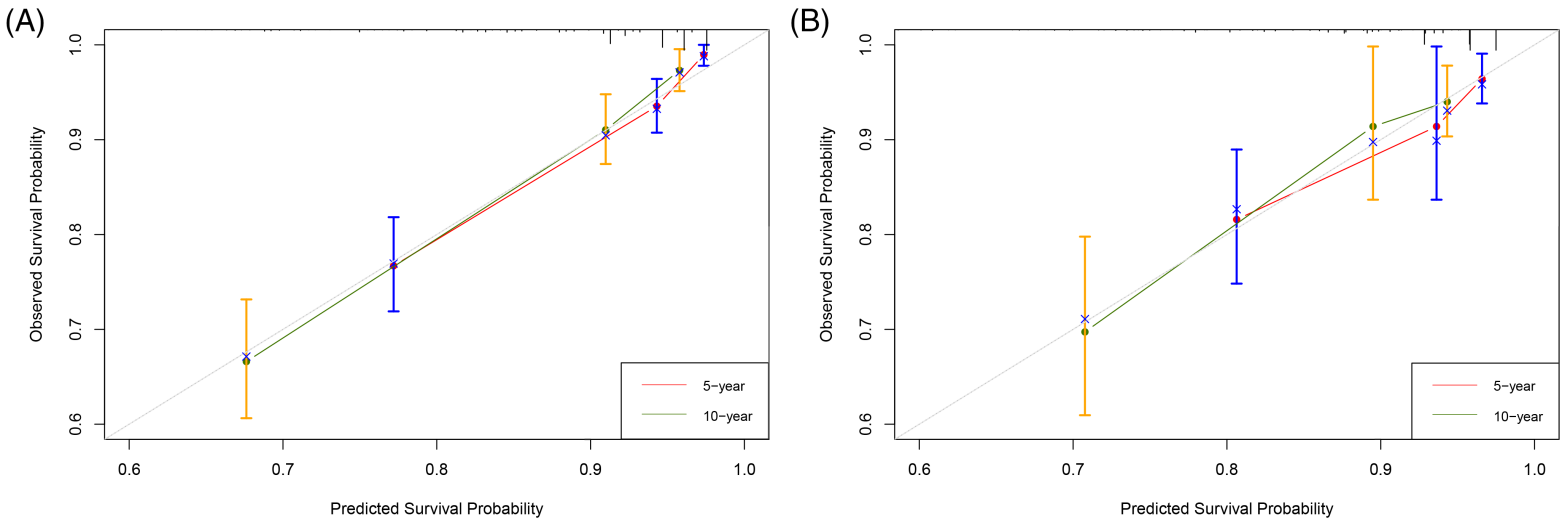
Calibration curves of the nomogram for 5‐ and 10‐year CSS in patients with OGCTs. (A) calibration curve for 5‐ and 10‐year CSS in the training set; (B) calibration curve for 5‐ and 10‐year CSS in the testing set. CSS, cancer‐specific survival; OCGTs, ovarian granulosa cell tumors.

The model's predictive power was gauged with Harrell's C‐index and time‐dependent receiver operating characteristic (ROC) curves. The Harrell's C‐indices for CSS were 0.798 (95% CI = 0.754–0.828) in the training set and 0.761 (95% CI = 0.698–0.824) in the testing set, respectively, both of which are greater than 0.75, which suggest a good discriminative ability of the model. Besides, the ROC curves showed that the area under ROC curves (AUCs) values for 5‐ and 10‐year CSS predictions in the training set were 0.82 and 0.79, respectively, and 0.75 and 0.80 in the testing set, respectively (shown in Figure 4). This also implies that the model has favorable distinguishing ability at 5 and 10 years. How well the predicted probabilities by the model matched the actual observed probabilities was assessed with calibration plots. The calibration plots in our study showed excellent consistency of the predicted and observed 5‐ and 10‐year CSS probability in both the training and testing sets (Figure 5), which confirmed our model's high accuracy between predicted value of the model and the actual observed value.

### 3.5 Clinical value of nomogram

**FIGURE 6:**
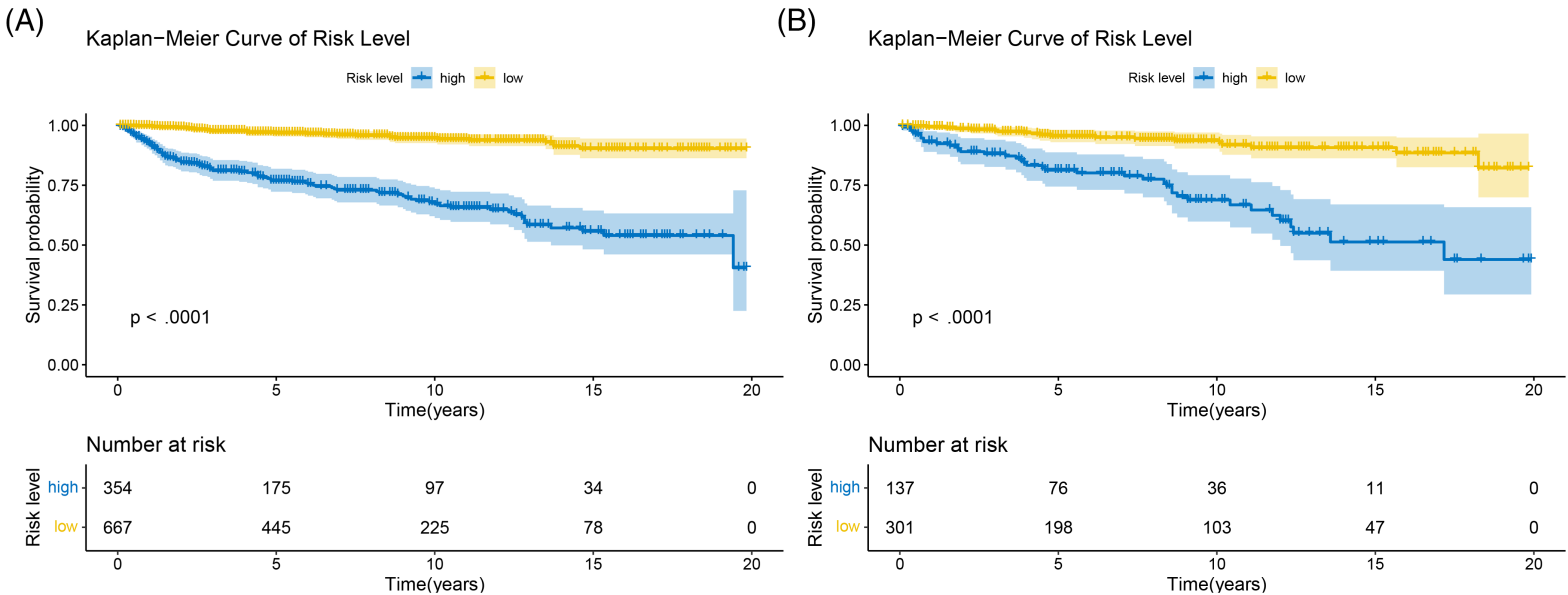
Kaplan–Meier survival curve in patients with OGCTs. (A) Kaplan–Meier survival curve in the training set. (B) Kaplan–Meier survival curve in the testing set. Patients in the training and testing sets were divided into high‐ and low‐risk groups with the same cut‐off values. The cut‐off value is the median risk score in the training set, which was calculated using the nomogram. Compared to the low‐risk group, the high‐risk group was linked to a higher likelihood of CSD (*p* < .001). CSD, cancer‐specific death; OGCTs, ovarian granulosa cell tumors.

**FIGURE 7:**
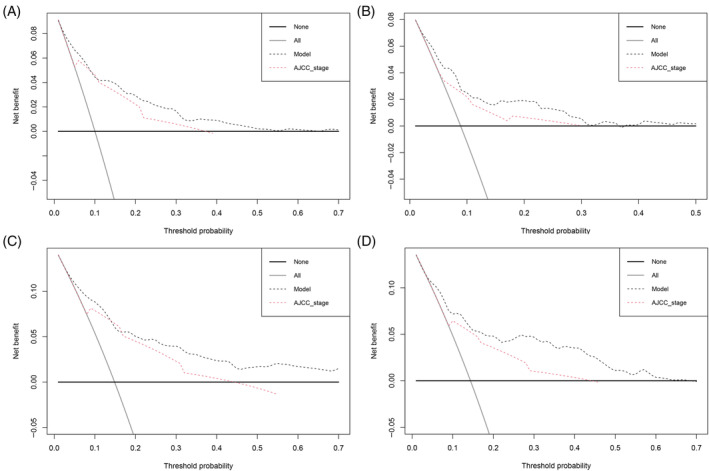
DCA regarding the nomogram and AJCC stage for patients with OGCTs. DCA for (A) 5‐year and (B) 10‐year CSS in the training set; DCA for (C) 5‐year and (D) 10‐year CSS in the testing set. AJCC, American Joint Committee on Cancer; CSS, cancer‐specific survival; DCA, decision curve analysis; OGCTs, ovarian granulosa cell tumors.

To evaluate how feasible and valid the prediction model was, prognostic scores for all patients in the cohort were obtained according to the model. The participants from both training and testing sets were stratified into low‐ and high‐risk groups using the same cut‐off value and the median prognostic scores in the training set. The Kaplan–Meier survival curves revealed that CSS for the low‐risk and high‐risk groups in both sets were significantly different (Figure 6), which further indicated the model's great discrimination. To further explore the clinical significance of the model in a clinical setting, we made the DCA plot to assess NB at different threshold probabilities, which displayed high values of NB for the model across a wide range of threshold probabilities in both the training and testing sets (Figure 7). By comparing the NB for the predictive model and that for AJCC staging system, we found that the model has a higher NB than AJCC staging system in both sets. We therefore conclude that the predictive model can be applied in clinical practice across the range of thresholds and presents superior value to AJCC staging system in clinical decision‐making.

## 4 DISCUSSION

The incidence of OGCTs is around 0.47–1.6 per 100 000 women worldwide per year.^18^ Although OGCTs present low malignant potential with a favorable prognosis, 20%–25% of patients ultimately experienced recurrence or metastasis and 70%–80% of them died from the disease.^19^, ^20^, ^21^ The 5‐ and 10‐year survival rates for OGCTs have been shown to be 98% and 96%, respectively. Our current study utilized univariate and multivariate Cox proportional risk regression models to identify independent predictive factors for CSS in patients with OGCTs, including age at diagnosis, pTNM stage, tumor size, surgery of primary tumor, surgery of regional LNs, residual disease after surgery, and chemotherapy. These factors, along with the results of LASSO regression analysis and clinical significance, led to the selection of age at diagnosis, pTNM stage, tumor size, surgery of primary tumor, and residual disease after surgery to build a predictive nomogram for 5‐ and 10‐year CSS of patients with OGCTs. The nomogram experienced validation with testing set and showed favorable discrimination and calibration, but also more predictive power than did the AJCC staging system. Furthermore, an online calculator (https://luochengyan66.shinyapps.io/dynnomapp/) was designed for clinicians' easier use on patient counseling and personalized treatment.

Despite that, many studies have explored the prognostic predictors of OGCTs recurrence, limited data are available in the prediction of OGCTs specific survival. Consequently, in our study of 1459 patients with OGCTs from SEER database, age at diagnosis, pTNM stage, tumor size, surgery of primary tumor, surgery of regional LNs, residual disease after surgery, and chemotherapy were demonstrated as independent predictors for CSS. Of these factors, tumor stage was the only universally recognized prognostic factor for the recurrence and OS in OGCTs patients, which was also confirmed by our study.^6^, ^17^, ^22^, ^23^, ^24^ The multivariate cox regression analysis in the current study revealed that increased T stage (stage T2, HR = 1.71, 95% CI = 1.12–2.62, *p* = .013; stage T3, HR = 2.55, 95% CI = 1.72–3.79, *p* < .001), with LNs metastasis (HR = 1.93, 95% CI = 1.03–3.63, *p* = .041) and with distant metastasis (HR = 2.14, 95% CI = 1.37–3.34, *p* < .001) were independent risk factors and correlated to worse CSS for patients with OGCTs, which was in accordance with previous studies.^20^, ^24^, ^25^, ^26^, ^27^, ^28^ According to Seagle et al, the 5‐year CSS rates for patients with stage I, II, III, and IV OGCTs were 98.2%, 89.3%, 81.0%, and 66.7%, respectively, with the 10‐year OS rate ranging from 85% to 95%, and disease mortality rate being about 20%.^17^ In addition to the aforementioned parameters, patients with older age (>64 years), tumor size (>99 mm), the occurrence of residual disease after surgery, and adjuvant chemotherapy presented poor CSS, whereas those undergone surgery of primary tumor and surgery of regional LNs exhibited improved CSS according to our current exploration. Our results demonstrated general consistency with those in the previous studies. A retrospective study including 2680 women with OGCTs from the National Cancer Database suggested that older age, presence of comorbidities, prior malignancy, larger tumor size, incomplete surgical staging, and residual lesions at the surgical margins were independently correlated to the elevated hazard of death.^17^ Ayhan et al found older age was a significant risk factor for both OS and CSS, with patients under 60 years of age surviving longer on average (154.6 vs. 89.2 months, *p* = 0.015).^29^ A multicenter study included 1426 patients with OGCTs and revealed that remaining disease after primary surgery (HR = 10.39, 95% CI = 3.15–34.29) and LNs metastasis (HR = 5.58, 95% CI = 1.62–19.19) were significant risk factors for CSS.^30^ Evidence supported similar roles for age and postoperative residual lesions as independent poor prognostic factors in the prognosis of OGCTs.^31^, ^32^ Tumor larger than 99 mm was predictive of poor CSS for patients with OGCTs according to the present study. The study by Seagle et al showed that each centimeter increase in tumor size increases the risk of death in stage I patients by 4% (*p* < .001).^17^ Results from Berek et al supported a similar role for tumor size in the prognosis of OGCTs.^33^ For newly diagnosed and recurrent OGCTs, the most effective treatment is to remove the primary tumor and metastatic lesions to the maximum extent.^7^, ^22^ Our results from the multivariate analysis supported the viewpoint and demonstrated that surgery of primary tumor and regional LNs improved CSS for OGCTs. With respect to LNs dissection, previous reports on some small‐scaled cohorts of patients with OGCTs undergoing lymphadenectomy (LND) have concluded that LNs dissection in OGCTs is unnecessary because of a very low occurrence of LNs metastasis.^19^, ^34^, ^35^ However, whether lymphadenectomy should be performed is still under controversy for the surgical management of OGCTs. According to a retrospective study by Erkilinç et al, in which 53 (53%) of 98 patients with OGCTs received pelvic‐paraaortic LND, no significant differences were found in disease‐free survival (DFS) and OS between women who did and did not undergo LND. To be notable, 92% patients in this study was at stage I and the rate of LN metastasis was only 3.1%.^36^ In a large‐scaled study on patients with stage I SCSTs by Nasioudis et al, the performance of LND was associated with worse OS.^37^ These results shows that LND exerts no benefits for those with stage I OGCTs. In our study, 634 (43.5%) women with OGCTs were performed LND and the stage distribution was as: 439 (69.24%) at stage I, 85 (13.4%) at stage II, 90 (14.19%) at stage III, and 20 (3.15%) at stage IV. In the whole cohort, patients who underwent LND had better CSS compared to those who did not. Therefore, our results suggested that LND was beneficial among women with OGCTs at all stages for the whole cohort, which accounts for the inconsistent results between our study and the previous studies. Of course, we need to further confirm this result by subgroup analysis in the future study. According to NCCN guidelines, platinum‐based chemotherapy is suggested to OGCTs patients with stage I with high‐risk factors (ruptured or poorly differentiated tumor), as well as those at stage II to IV with category 2B recommendations.^7^, ^8^, ^38^, ^39^ Based on the available current studies, the value of adjuvant chemotherapy is uncertain even in stage I with high‐risk factors (ruptured or poorly differentiated tumor), or advanced stage, or recurrent OGCTs without residual tumor after surgery.^17^, ^40^, ^41^, ^42^ Chemotherapy displayed unfavorable hazard ratio estimates for CSS of 1.48 (95% CI = 1.07–2.05, *p* = .018), and this may be attributed to the indolent nature of OGCTs, the patient's insensitivity to chemotherapy and toxicity of chemotherapeutic regimens. Comparable results were reported by You D et al with a population of 760 patients with OGCTs.^43^

Using the prognostic factors analyzed by multivariate Cox regression, the predictive power was analyzed by LASSO regression analysis in our study and clinical significance of the variables, age at diagnosis, tumor size, pTNM stage, surgery of primary tumor, and residual disease after surgery were incorporated in the nomogram. The quality of the model was gauged from the perspective of discrimination and calibration. C‐index and AUC values, the most commonly used indicators for discrimination, range from 0.5 to 1.0 and a larger value suggests better discrimination. In our study, the C‐indices and time‐dependent AUCs for both the training and testing cohorts were greater than 0.75, suggesting the model accurately predicts CSS in OGCTs patients. As our results demonstrate, the C‐indices and time‐dependent AUC values of the testing set is slightly lower than those of the training set, which may be related to the sample size of the validation set and the slightly smaller number of women who experienced OGCTs‐specific death events. We sincerely hope to improve the results by expanding the sample size in further studies. Calibration plots in the current study exhibited good concordance between the forecasted and observed 5‐ and 10‐years CSS probability in both the training and testing cohorts, which indicate our model was well‐calibrated. To investigate the usefulness of our model, DCA was performed in both training and testing sets and higher NB for the model was proved, which indicated that this model can be used in clinical practice across the range of thresholds and presents superior value to AJCC staging system in clinical decision‐making. Therefore, the nomogram is a reliable tool for accurately predicting the 5‐ and 10‐year CSS of OGCTs patients and is more valuable than AJCC staging systems for practical decision‐making. By using the predictive model and nomograms, clinicians can calculate the risk scores based on clinicopathological characteristics of OGCT patients, identify at‐risk populations, and guide treatment, determine prognosis, and make personalized follow‐up decisions. As noted above, there is uncertainty about the efficacy of chemotherapy for OGCTs. In clinical practice, based on this predictive model and risk score, a patient who is concluded to be at high‐risk can be given chemotherapy after communication and informed consent to improve prognosis. So far as we know, ours is the first nomogram with biggest cohort to predict CSS for patients with OGCTs. After searching for the existing literature, only one nomogram on recurrence free survival of OGCTs was developed with moderate discrimination (C‐index = 0.73, 95% CI = 0.66–0.80) and calibration.^4^ By using SEER database, You D et al enrolled 913 patients with ovarian SCSTs and established a nomogram to predict OS.^43^ In this cohort, a total of 760 (83.2%) women were with OGCTs. To date, no prognostic models were developed predicting the CSS for women with OGCTs. Based on the prediction model, the present study not only displayed the formula to allow for future external validation and further optimization, but also quantified the predictive parameters with static nomogram and established a web‐based calculator to obtain 5‐ and 10‐ year CSS for patients with OGCTs.

Our present study surely carries certain limitations. First, its retrospective nature and the presence of missing values with some parameters (such as tumor size and residual disease after surgery) might cause bias. Due to the rarity and indolent nature of the disease, it is difficult to perform prospective randomized controlled trial and to achieve statistical power. The SEER database provides sufficient patient information and complete follow‐up information for malignancies, including OGCTs, and is therefore an important source of research on this relatively rare disease, for example, model construction and validation, which can provide an important guide for clinicians. Secondly, the information retrieved from SEER database spans about 20 years and the disease management including surgical approach and chemotherapy regimens have changed, which might affect the prognosis. Lastly, although the nomogram shows good performance in internal validation, the biological heterogeneity of the two histological subtypes of OGCTs (aOGCTs and jOGCTs) limits the generalization and application of the nomogram. We will expand the sample size to confirm the findings and externally verify it in the further study by using independent cohorts.

## 5 CONCLUSION

In conclusion, age at diagnosis, pTNM stage, tumor size, surgery of primary tumor, surgery of regional LNs, residual disease after surgery, and chemotherapy were revealed to be independent predictors for CSS in OGCT patients according to our current study. By combining the predictive power and clinical significance of the variables, we constructed the nomogram with age, tumor size, pTNM stage, surgery of primary tumor, and residual disease after surgery, and compared with the AJCC staging system, our nomogram showed favorable discrimination, accurate calibration, and more powerful predictive value. The nomogram was displayed in the form of a stationary nomogram and a web‐based calculator. It facilitates clinicians to individualize optimal treatment and follow‐up. Multicenter clinical trials are warranted in the future to externally validate this predictive model.

## AUTHOR CONTRIBUTIONS

**Yue Zhang:** Data curation (equal); writing – original draft (equal); writing – review and editing (equal). **Zhen Zhang:** Data curation (equal); writing – original draft (equal); writing – review and editing (equal). **Xinyao Ding:** Data curation (equal); writing – original draft (equal); writing – review and editing (equal). **Keyi Zhang:** Formal analysis (equal). **Youren Dai:** Formal analysis (equal). **WenJun Cheng:** Formal analysis (equal). **Chengyan Luo:** Conceptualization (lead); funding acquisition (lead); methodology (lead); project administration (lead); supervision (lead).

## FUNDING INFORMATION

Project of Maternal and Child Health of Jiangsu, China. Grant/Award Number: F202118.

## CONFLICT OF INTEREST STATEMENT

The authors have stated explicitly that there are no conflicts of interest in connection with this article.

## ETHICS STATEMENT

The study conformed to the Declaration of Helsinki (2013). The approval from the institutional review board is waived because the SEER database is a public database with open accessibility. Since this retrospective study is based on public data available in the SEER database, no human subjects or personal privacy is involved. Therefore, informed consent from patients is not required.

## Supporting information


**Figure S1.** Least absolute shrinkage and selection operator (LASSO) regression analysis via 10‐fold cross‐validation. (A) LASSO coefficient profiles of the 19 variables. (B) A 10‐fold cross‐validation results. The left dotted line represents the optimal values with the minimum criteria and right dotted line represents one standard error criterion. As the value of λ reduced, the degree of model compression increased and the function of powerful variables selection strengthened.

**Figure S2.** The web‐based nomogram being displayed as an online calculator for predicting the probability of CSS in OGCTs patients. The screenshots from the web shows the 5‐ and 10‐year probability of CSS based on the predictive model was 0.68 (95% CI: 0.58–0.80) and 0.54 (95% CI: 0.42–0.69), respectively, for a 37‐year‐old OGCTs patient with stage T3, N0, M0, tumor size of 130 mm, undergoing surgery of primary site and with residual disease after surgery. CSS, cancer‐specific survival; OGCTs, ovarian granulosa cell tumors.

**Figure S3.** Flowchart of the research design and data analysis. DCA, decision curve analysis; LASSO, Least absolute shrinkage and selection operator; ROC, receiver operator characteristic.

**Table S1.** Basic characteristics of patients with OGCTs from the SEER database after multiple imputation. AJCC, American Joint Committee on Cancer; CSS, cancer‐specific survival; LNs, lymph nodes; OGCTs, ovarian granulosa cell tumors; SEER, Surveillance, Epidemiology, and End Result; stage M, stage of metastasis; stage N, stage of lymph nodes; stage T, stage of primary tumor.

## Data Availability

The datasets used and/or analyzed during the current study are available from the corresponding author on reasonable request.
