# Supplementary figures and images for: Identification of prognostic factors and construction of nomogram to predict cancer‐specific survival for patients with ovarian granulosa cell tumors
Source: Cancer Rep (Hoboken). 2024 Mar 20;7(3):e2046. doi: 10.1002/cnr2.2046 (PMC10953832; doi:10.1002/cnr2.2046)

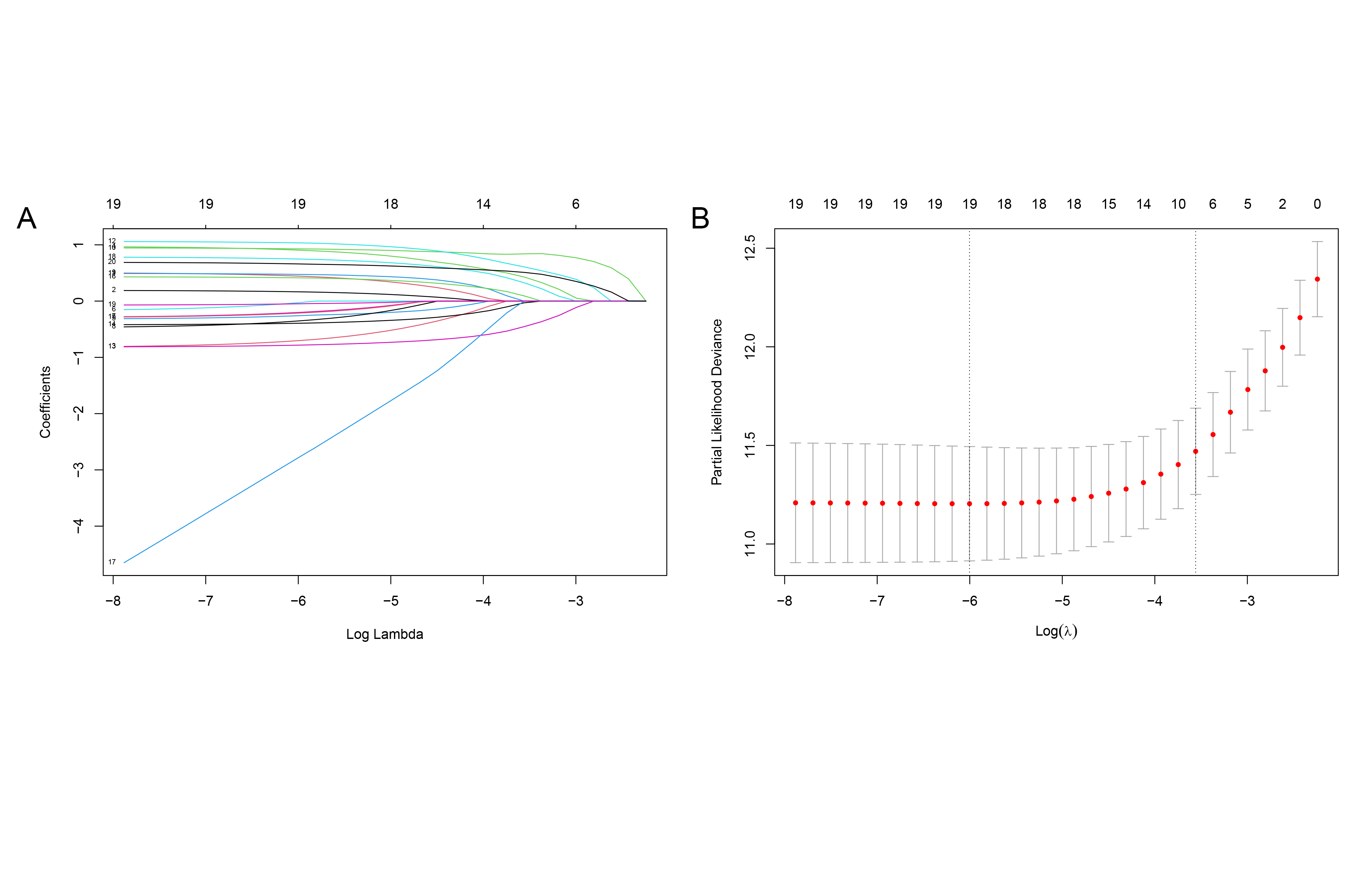

Supplement: Supplementary file 1 — Figure S1. Least absolute shrinkage and selection operator (LASSO) regression analysis via 10‐fold cross‐validation. (A) LASSO coefficient profiles of the 19 variables. (B) A 10‐fold cross‐validation results. The left dotted line represents the optimal values with the minimum criteria and right dotted line represents one standard error criterion. As the value of λ reduced, the degree of model compression increased and the function of powerful variables selection strengthened. [file CNR2-7-e2046-s004.tif]

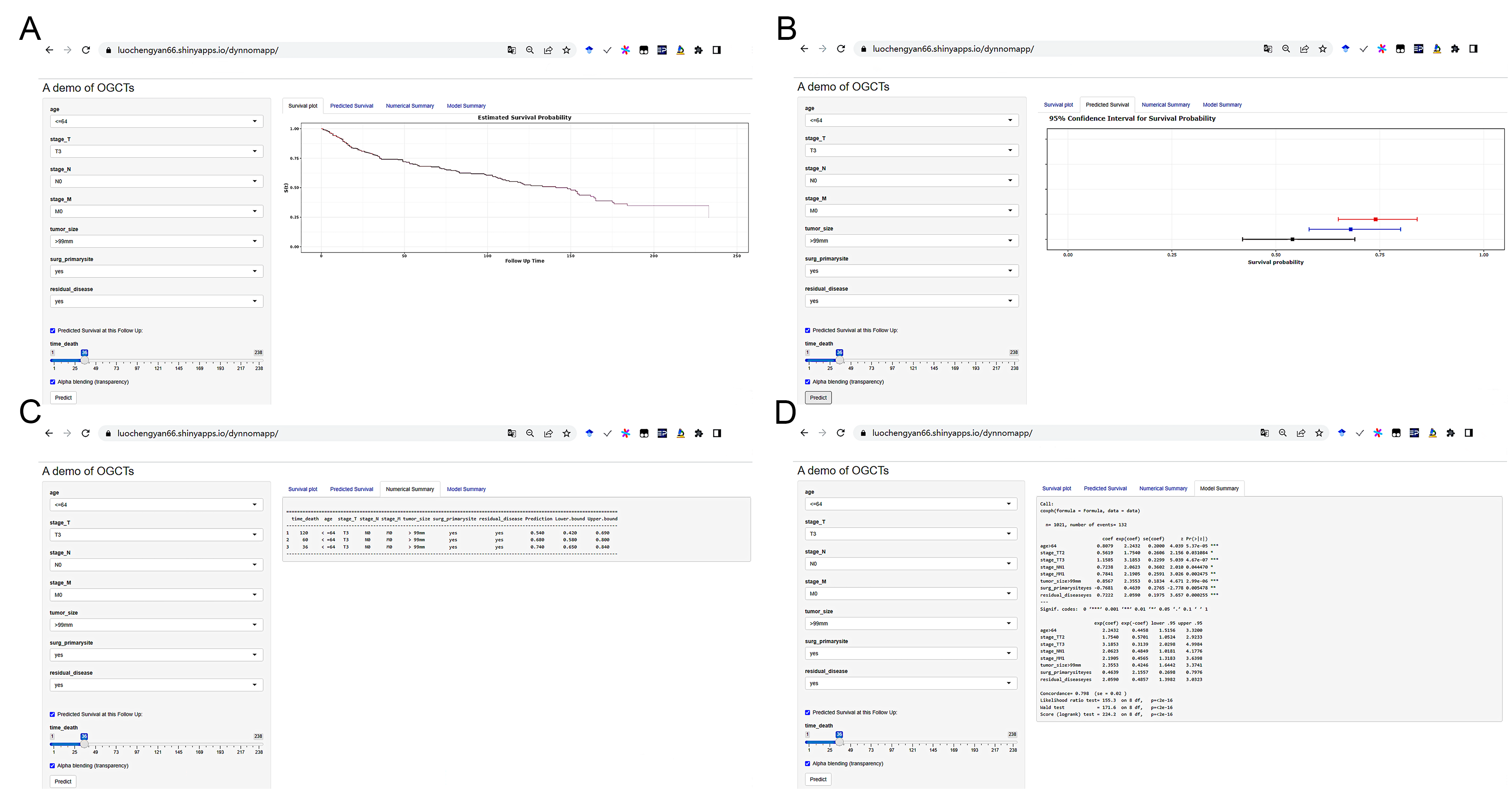

Supplement: Supplementary file 2 — Figure S2. The web‐based nomogram being displayed as an online calculator for predicting the probability of CSS in OGCTs patients. The screenshots from the web shows the 5‐ and 10‐year probability of CSS based on the predictive model was 0.68 (95% CI: 0.58–0.80) and 0.54 (95% CI: 0.42–0.69), respectively, for a 37‐year‐old OGCTs patient with stage T3, N0, M0, tumor size of 130 mm, undergoing surgery of primary site and with residual disease after surgery. CSS, cancer‐specific survival; OGCTs, ovarian granulosa cell tumors. [file CNR2-7-e2046-s003.tif]

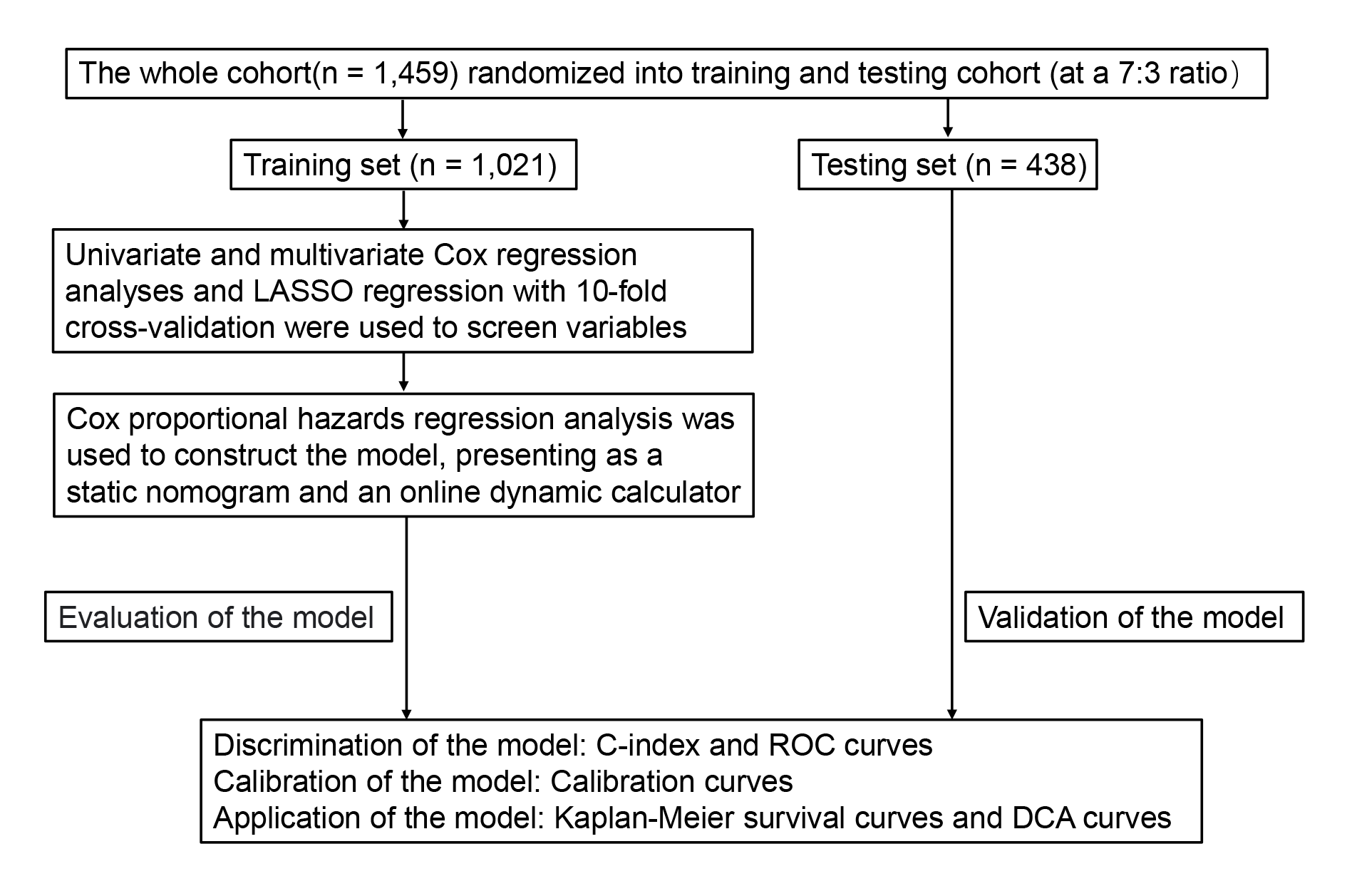

Supplement: Supplementary file 3 — Figure S3. Flowchart of the research design and data analysis. DCA, decision curve analysis; LASSO, Least absolute shrinkage and selection operator; ROC, receiver operator characteristic. [file CNR2-7-e2046-s001.tif]
