# Supplementary material for: Identification of prognostic factors and construction of nomogram to predict cancer‐specific survival for patients with ovarian granulosa cell tumors
Source: Cancer Rep (Hoboken). 2024 Mar 20;7(3):e2046. doi: 10.1002/cnr2.2046 (PMC10953832; doi:10.1002/cnr2.2046)
Supplement: Supplementary file 4 — Table S1. Basic characteristics of patients with OGCTs from the SEER database after multiple imputation. AJCC, American Joint Committee on Cancer; CSS, cancer‐specific survival; LNs, lymph nodes; OGCTs, ovarian granulosa cell tumors; SEER, Surveillance, Epidemiology, and End Result; stage M, stage of metastasis; stage N, stage of lymph nodes; stage T, stage of primary tumor. [file CNR2-7-e2046-s002.docx]

**Table S1**. Basic characteristics of patients with OGCTs from the SEER database after multiple imputation.

| **Variable** | **Training set**  **(N=1,021)** | **Testing set**  **(N=438)** | **P value** |
| --- | --- | --- | --- |
| **Age (years)** |  |  | 0.768 |
| ≤64 | 831 (81.4%) | 360 (82.2%) |  |
| >64 | 190 (18.6%) | 78 (17.8%) |  |
| **Race (%)** |  |  | 0.459 |
| White | 696 (68.2%) | 295 (67.4%) |  |
| Black | 240 (23.5%) | 98 (22.4%) |  |
| other | 85 (8.3%) | 45 (10.3%) |  |
| **Marital status (%)** |  |  | 0.658 |
| Sigle | 261 (25.6%) | 116 (26.5%) |  |
| Married or ever married | 705 (69.0%) | 294 (67.1%) |  |
| unknown | 55 (5.4%) | 28 (6.4%) |  |
| **Multi-primary tumors (%)** |  |  | 0.547 |
| One primary only | 839 (82.2%) | 359 (82.0%) |  |
| 1st of 2 or more primaries | 80 (7.8%) | 29 (6.6%) |  |
| 2nd or more of primaries | 102 (10.0%) | 50 (11.4%) |  |
| **AJCC stage (%)** |  |  | 0.858 |
| I | 689 (67.5%) | 306 (69.9%) |  |
| II | 128 (12.5%) | 52 (11.9%) |  |
| III | 146 (14.3%) | 58 (13.2%) |  |
| IV | 58 (5.7%) | 22 (5.0%) |  |
| **pT stage (%)** |  |  | 0.644 |
| T1 | 708 (69.3%) | 314 (71.7%) |  |
| T2 | 142 (13.9%) | 54 (12.3%) |  |
| T3 | 171 (16.7%) | 70 (16.0%) |  |
| **pN stage (%)** |  |  | 0.941 |
| N0 | 993 (97.3%) | 427 (97.5%) |  |
| N1 | 28 (2.7%) | 11 (2.5%) |  |
| **pM stage (%)** |  |  | 0.650 |
| M0 | 962 (94.2%) | 416 (95.0%) |  |
| M1 | 59 (5.8%) | 22 (5.0%) |  |
| **Surgery of primary tumor (%)** |  |  | 0.417 |
| no | 59 (5.8%) | 20 (4.6%) |  |
| yes | 962 (94.2%) | 418 (95.4%) |  |
| **Surgery of regional LNs (%)** |  |  | 0.477 |
| no | 584 (57.2%) | 241 (55.0%) |  |
| yes | 437 (42.8%) | 197 (45.0%) |  |
| **Surgery of distant metastasis (%)** |  |  | 0.552 |
| no | 919 (90.0%) | 389 (88.8%) |  |
| Yes | 102 (10.0%) | 49 (11.2%) |  |
| **Chemotherapy (%)** |  |  | 0.385 |
| no | 719 (70.4%) | 319 (72.8%) |  |
| yes | 302 (29.6%) | 119 (27.2%) |  |
| **Radiation (%)** |  |  | 0.566 |
| no | 1008 (98.7%) | 430 (98.2%) |  |
| yes | 13 (1.3%) | 8 (1.8%) |  |
| **Tumor size (mm)** |  |  | 0.896 |
| ≤99 | 519 (50.8%) | 225 (51.4%) |  |
| >99 | 502 (49.2%) | 213 (48.6%) |  |
| **Residual disease after surgery (%)** |  |  | 0.140 |
| no | 881 (86.3%) | 391 (89.3%) |  |
| yes | 140 (13.7%) | 47 (10.7%) |  |
| **CSS** |  |  | 0.938 |
| Alive | 889 (87.1%) | 380 (86.8%) |  |
| OGCTs specific death | 132 (12.9%) | 58 (13.2%) |  |

OGCTs, ovarian granulosa cell tumors; SEER, Surveillance, Epidemiology, and End Results; AJCC, American Joint Committee on Cancer; pT stage, stage of primary tumor; pN stage, stage of lymph nodes; pM stage, stage of metastasis; LNs, lymph nodes; CSS, cancer-specific survival.
